# Development of a Triple-Color Pseudovirion-Based Assay to Detect Neutralizing Antibodies against Human Papillomavirus

**DOI:** 10.3390/v8040107

**Published:** 2016-04-25

**Authors:** Jianhui Nie, Yangyang Liu, Weijin Huang, Youchun Wang

**Affiliations:** Division of HIV/AIDS and Sexually Transmitted Virus Vaccines, National Institutes for Food and Drug Control (NIFDC), No. 2 Tiantanxili, Beijing 100050, China; niejhfirst@126.com (J.N.); yangyangliu0102@163.com (Y.L.); Huangweijin@nifdc.org.cn (W.H.)

**Keywords:** human papillomavirus, HPV, vaccine, FluoroSpot, multiplex immunoassay

## Abstract

Pseudovirion-based neutralization assay is considered the gold standard method for evaluating the immune response to human papillomavirus (HPV) vaccines. In this study, we developed a multicolor neutralization assay to simultaneously detect the neutralizing antibodies against different HPV types. FluoroSpot was used to interpret the fluorescent protein expression instead of flow cytometry. The results of FluoroSpot and flow cytometry showed good consistency, with R^2^ > 0.98 for the log-transformed IC_50_ values. Regardless of the reporter color, the single-, dual-, and triple-color neutralization assays reported identical results for the same samples. In low-titer samples from naturally HPV-infected individuals, there was strong agreement between the single- and triple-color assays, with kappa scores of 0.92, 0.89, and 0.96 for HPV16, HPV18, and HPV58, respectively. Good reproducibility was observed for the triple-color assay, with coefficients of variation of 2.0%–41.5% within the assays and 8.3%–36.2% between the assays. Three triple-color systems, HPV16-18-58, HPV6-33-45, and HPV11-31-52, were developed that could evaluate the immunogenicity of a nonavalent vaccine in three rounds of the assay. With the advantages of an easy-to-use procedure and less sample consumption, the multiple-color assay is more suitable than classical assays for large sero-epidemiological studies and clinical trials and is more amenable to automation.

## 1. Introduction

Cervical cancer is the second most frequently diagnosed cancer and the third leading cause of cancer death among women in developing countries [1], and is strongly associated with high-risk human papillomavirus (HPV) infection. To date, more than 200 HPV genotypes have been identified [2], among which 15 are classified as high-risk types (HPV16, 18, 31, 33, 35, 39, 45, 51, 52, 56, 58, 59, 68, 73, and 82) [3,4]. HPV vaccines are an effective prophylactic strategy implemented in many countries, and a bivalent HPV type 16/18 vaccine (Cervarix^®^) and a quadrivalent HPV type 6/11/16/18 vaccine (Gardasil^®^) have been licensed for use [3,5]. In clinical trials, the vaccines have shown almost 100% efficacy against precancerous lesions associated with the vaccine HPV types, which account for 70% of cervical cancer cases worldwide [3]. In 2014, a nonavalent HPV vaccine, Gardasil 9, directed against HPV6, 11, 16, 18, 31, 33, 45, 52, and 58, was approved by the U.S. Food and Drug Administration (FDA), increasing the potential prevention of cervical cancer from 70% to 90% [6].

In HPV vaccinology and epidemiology, serological testing methods are used to analyze the antibody levels in naturally infected or vaccinated individuals. A variety of methods have been developed for testing antibody levels, including the competitive Luminex^®^ immunoassay (cLIA) [7], the virus-like-particle (VLP)-based multiplex immunoassay (VLP-MIA) [8], and the *in situ* purified glutathione S-transferase L1-based MIA (GST-L1-MIA) [9]. Depending on the preselected monoclonal antibody, the cLIA results are interpreted to the overlapping epitopes, which might not predict the *in vivo* protection well when the dominant antibodies in some serum samples are not the selected epitopes. VLP-MIA and GST-L1-MIA detect the total VLP-specific antibodies, which include some non-neutralizing antibodies, and might therefore overestimate the efficacy of the vaccine.

Although the level of protection afforded by neutralizing antibodies has not been determined, neutralizing antibodies are accepted as the primary mediator of a vaccine’s potency. The HPV life cycle is strictly dependent on the differentiation stage of the host cell [10]. Native HPV virions cannot be produced in conventional culture, and it is almost impossible to detect neutralizing antibodies using authentic virions, especially in large-scale analyses of naturally infected or vaccinated cohorts. The cotransfection of mammalian cells with two HPV capsid genes, L1 and L2, together with a reporter plasmid produced high infectious titers of pseudovirions, which presented surface conformational epitopes similar to those of the native virions [11]. The pseudovirion-based neutralization assay (PBNA) is recognized as the gold standard method for the analysis of the functional humoral immune response to HPV. Several PBNAs have been developed using different reporter genes, including fluorescent reporter genes (such as enhanced green fluorescent protein (EGFP) [12,13]), or chemiluminescent reporter genes (such as secreted alkaline phosphatase (SEAP) [12] and *Gaussia* luciferase (Gluc) [14,15]). The infected status of the target cells in the PBNA is detected using fluorescent microscopy and/or flow cytometry (FCM) for EGFP and with a chemiluminescence reader for SEAP and GLuc. Because the interpretation with microscopy is subjective and the procedure for FCM is laborious, the EGFP method has not been widely used. Although the SEAP- and GLuc-based method can enhance the throughput to a certain extent, the throughput is still lower than that of MIAs [7,16], which can simultaneously quantitate antibodies directed against different HPV types. Recent advances in fluorescent reporter genes and the ELISPOT reader allow the simultaneous detection of several differently colored fluorescent proteins [17]. Using these innovations, we have established a new multicolor PBNA to simultaneously quantify the neutralizing antibodies directed against different types of HPV.

## 2. Materials and Methods

### 2.1. Cells, Plasmids, Serum Samples, HPV Antibody Standards, and HPV Vaccines

The 293FT cell line (Invitrogen, Carlsbad, CA, USA) was maintained in growth medium (high-glucose Dulbecco’s modified Eagle’s medium with 10% fetal bovine serum, 1% penicillin-streptomycin solution, 1% nonessential amino acids, 2% HEPES). HPV L1/L2 expressing plasmids (p16LLw [18], p18LLw [12], p6LLw2 [19], p11Lw [20], p11∫w [21], p31sheLL [22], p45sheLL [23], p52LLw [24] and p58LLw [22]) and the red fluorescent protein (RFP) reporter plasmid pRwB [25] were kindly provided by John Schiller (National Cancer Institute, Bethesda, MD, USA). E2-CFP (Clontech, Mountain View, CA, USA) expresses the E2-crimson fluorescent protein (CFP). The EGFP reporter plasmid was constructed by inserting the *EGFP* gene into the pCDNA3.1 vector (Invitrogen, Carlsbad, CA, USA), as described previously [26].

### 2.2. Serum Samples

Naturally HPV-infected serum samples were selected from a large number of samples from a blood bank (Shanghai RAAS Blood Products Co. Ltd, Shanghai, China). Fifty post-vaccinated human serum samples that had been collected in a phase I clinical trial of a bivalent HPV16/18 vaccine, produced in *Pichia pastoris* (ClinicalTrials.gov ID: 2011L01085) were kindly provided by Shanghai Zerun Biotechnology (Shanghai, China). Written informed consent was obtained from all donors. Mouse sera were collected from mice intraperitoneally administered a candidate nonavalent HPV vaccine (Innovax, Xiamen, China). Rabbit serum samples were collected from rabbits immunized with a monovalent vaccine kindly provided by Qiming Li (National Vaccine and Serum Institute, Beijing, China), as described previously [15]. This study was approved by the Institutional Animal Care and Use Committee of the National Institutes for Food and Drug Control, China. All animals were housed and maintained in accordance with the relevant guidelines and regulations.

### 2.3. Preparation and Titration of HPV Pseudovirions

HPV pseudovirions were produced in 293FT cells using a previously described method, with modification [11,27]. Briefly, 293FT cells were cotransfected with HPV-L1/L2-expressing plasmids together with a reporter plasmid, using Lipofectamine 2000 (Invitrogen), according to the manufacturer’s instructions. The cells were harvested 48 h post-transfection and suspended in Dulbecco’s phosphate-buffered saline (Invitrogen) supplemented with 0.5% Triton X-100, 0.1% Benzonase^®^ (Sigma-Aldrich, St. Louis, MO, USA), 0.1% Plasmid-Safe ATP-Dependent DNase (Epicentre, Madison, WI, USA), and 1/40 volume of 1 M ammonium sulfate. The pseudovirions were matured by incubating the cell lysates overnight at 37 °C. The lysates were then transferred to an ice bath for 5 min and the NaCl concentration was adjusted to 0.85 M. The lysates were clarified by centrifugation at 5000× *g* for 10 min. The three types of pseudovirions (expressing reporter protein EGFP, RFP, or E2-CFP) were stored as aliquots at −80 °C until titration.

The pseudovirion stocks were titrated as follows: 293FT cells were trypsinized and placed in the inner wells of 96-well tissue culture plates at 1.5 × 10^4^ cells/100 μL/well for 3–6 h before the pseudovirions were added. Each type of pseudovirion was prediluted 100-fold, and nine serial five-fold dilutions were prepared. After incubation for 72 h at 37 °C under 5% CO_2_, the percentages of fluorescence-positive cells and fluorospots were determined with FCM (BD, Franklin Lakes, NJ, USA) and an ImmunoSpot reader (CTL, Shaker Heights, OH, USA). The pseudoviron titers were defined as 50% tissue culture infective dose (TCID50), calculated with the Reed-Muench method [28].

### 2.4. Pseudovirion-Based Neutralization Assay

Once the pseudovirion titer had been determined, the neutralizing antibody titers of the sera were determined with a PBNA, as in previous studies [12,13,15]. Briefly, 293FT cells were placed in 96-well flat-bottom cell culture plates at 15,000 cells/well with 100 μL of growth medium and incubated for 3–6 h before the pseudovirions were added. The pseudovirion stocks were diluted to 4000–6000 TCID50/mL [13]. The diluted pseudovirions (60 μL) and serially diluted sera (60 μL) were mixed in 96-well round-bottom plates and placed on ice for 1 h. The pseudovirion-serum mixtures (100 μL) were transferred into cell culture plates preseeded with 293FT cells, and incubated for 68–72 h. After incubation, the numbers of fluorospots were counted with the ImmunoSpot reader. The serum neutralization titers were defined as the 50% maximal inhibitory concentration (IC_50_), calculated with the Reed-Muench method [28].

## 3. Results

### 3.1. Selection of Fluorescent Proteins

**Figure 1:**
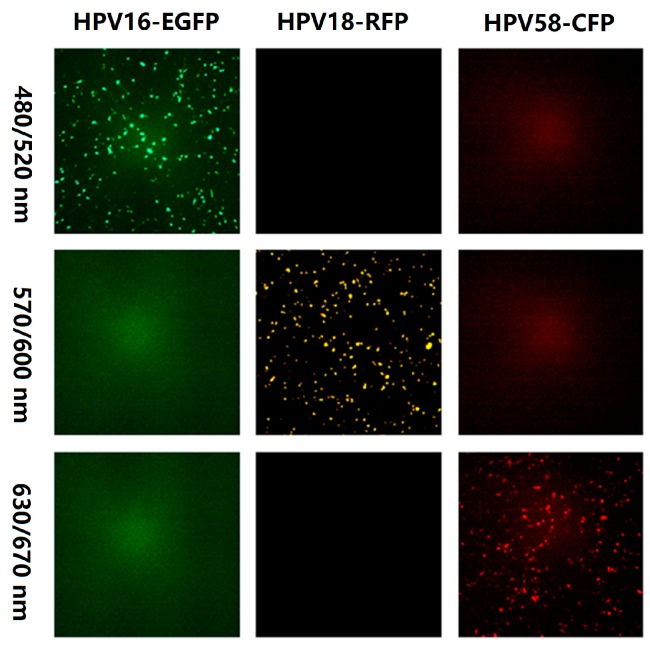
Fluorescence of three fluorescent proteins under different excitation and emission filters. The excitation and emission filter wavelengths for row 1 were 480 nm and 520 nm, respectively. For row 2, the excitation and emission filter wavelengths were 570 nm and 600 nm, respectively. For row 3, the excitation and emission filter wavelengths were 630 nm and 670 nm, respectively.

To select the appropriate fluorescent proteins for the multiplex PBNA, 293FT cells were transfected with different fluorescence-expressing plasmids: AmCyan1 (blue), EGFP (green), enhanced yellow fluorescent protein (EYFP; yellow), RFP (red), and E2-CFP (far red). The cells were scanned with different excitation and emission wavelengths using ImmunoSpot (CTL, Shaker Heights, OH, USA). Only EGFP, RFP, and E2-CFP were detected with unique excitation-emission wavelength pairs. Therefore, the three reporter plasmids were cotransfected with the HPV structural genes to generate pseudovirions producing different colors (Figure 1). No mutual interference was observed with the specific excitation-emission wavelength pairs used to detect the pseudovirions.

### 3.2. Correlation of the FluoroSpot and FCM Results

**Figure 2:**
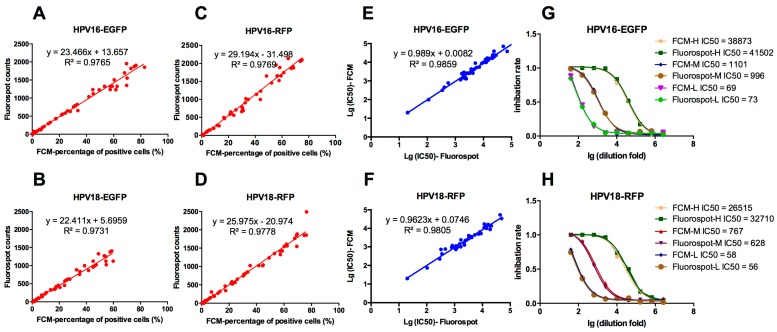
Correlation between FluoroSpot and FCM analyses of EGFP- and RFP-PBNA. (**A**–**D**) Correlation of the Fluorospot counts and FCM-percentage of positive cells when tested for HPV16-EGFP, HPV18-EGFP, HPV16-RFP and HPV18-RFP, respectively; (**E**,**F**) correlation of the lg(IC50) values for Fluorospot counts and FCM-percentage of positive cells when tested for HPV16-EGFP and HPV18-RFP, respectively; (**G**,**H**) the titration of three serum samples (H, M, and L with high, medium, and low neutralizing antibody titers collected from the dual-valent vaccine clinical tiral) against HPV16-EGFP and HPV18-RFP, respectively. The sensitivity parameters used in these assay was 215.

The classic GFP-pseudovirion assay was detected using the FCM [12]. To compare the results of FluoroSpot and FCM, 293FT cells were transfected with an HPV16 or HPV18 structural genes, together with the reporter gene *EGFP* or *RFP*, to yield four pseudovirions: HPV16-EGFP, HPV18-EGFP, HPV16-RFP, and HPV18-RFP. The 293FT cells were then infected with the serially diluted pseudovirions and detected with FluoroSpot and FCM 72 h post-infection. The FluoroSpot counts and the percentages of positive cells correlated well, with R^2^ > 0.97 (Figure 2A–D). The neutralizing antibodies against HPV16-EGFP and HPV18-RFP in 50 post-vaccination human serum samples were detected with both FluoroSpot and FCM. The log-transformed IC_50_ values showed good consistency, with R^2^ > 0.98 (Figure 2E,F).

### 3.3. Impact of the Sensitivity Parameter on the Interpretation of the FluoroSpot Results

**Figure 3:**
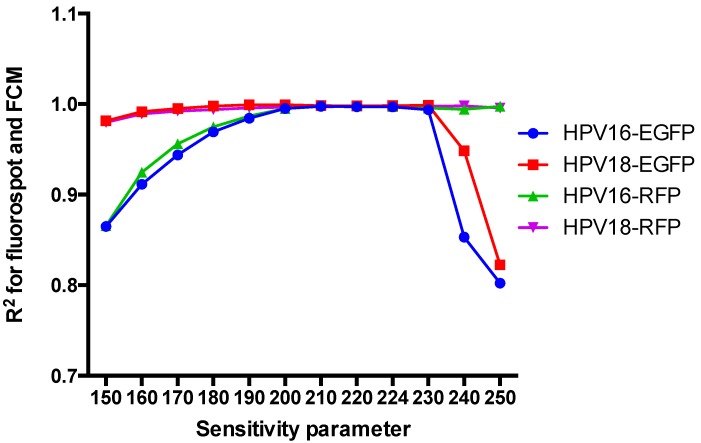
Effect of the sensitivity parameter on the correlation between the FluoroSpot and FCM results.

The sensitivity of the FluoroSpot counts can be adjusted by changing the sensitivity parameter. Too high a sensitivity could yield high background and low specificity, whereas too low a sensitivity might miss specific spots. To identify the optimal sensitivity parameter, 293FT cells were infected with HPV16-EGFP, HPV18-EGFP, HPV16-RFP, or HPV18-RFP and detected with different sensitivity parameters (150–250), and the results were compared with the FCM results. The linear correlation R^2^ increased initially and then decreased as the sensitivity parameter increased (Figure 3). The correlation was most easily disturbed by altering the sensitivity parameter for pseudovirions containing EGFP. Only when the sensitivity parameter was 200–230, the R^2^ reached more than 0.99. For the pseudovirions expressing RFP, R^2^ > 0.99 when the sensitivity parameter was 180–250. Therefore, 215 was used as the optimal sensitivity parameter for this assay. At this sensitivity, 300 and 400 fluorescent spots were detected for EGFP and RFP, respectively, if the rate of positive cells reached 15%, which was the most suitable pseudoviral dose for FCM [13].

### 3.4. Comparison of Single- and Dual-Color PBNA

**Figure 4:**
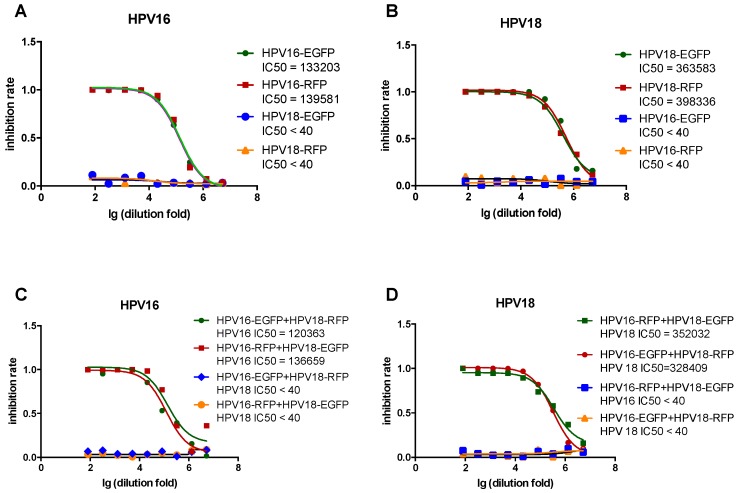
Comparison of single- and dual-color PBNA in detecting inhibition rates. (**A**) Single-color HPV16 and HPV18 pseudovirions were tested against mono-valent HPV16 vaccine immunized rabbit serum; (**B**) Single-color HPV16 and HPV18 pseudovirions were tested against monovalent HPV18 vaccine immunized rabbit serum; (**C**) Dual-color HPV16 and HPV18 pseudovirions mixtures were tested against monovalent HPV16 vaccine immunized rabbit serum; (**D**) Dual-color HPV16 and HPV18 pseudovirion mixtures were tested against monovalent HPV18 vaccine immunized rabbit serum.

To determine whether the reporter protein affects the PBNA results, green (EGFP) and red (RFP) HPV16 pseudovirions were tested against rabbit sera immunized with HPV16. The almost totally overlapping inhibition curves yielded similar IC_50_ values for HPV16 with both reporter proteins (Figure 4A). A similar phenomenon was observed for HPV18 (Figure 4B). To investigate whether the mixed pseudovirions affected the PBNA results, the same serum samples were tested with two dual-color pseudovirion combinations (HPV16-EGFP + HPV18-RFP and HPV16-RFP + HPV18-EGFP) (Figure 4C,D). The similar inhibition curves and IC_50_ values indicated that there was no distinct difference between these two combinations. The results for dual-color PBNA were also consistent with those for single-color PBNA (Figure 4). In addition, no cross-talk was observed between HPV16 and HPV18 in all the PBNAs.

### 3.5. Comparison of Single- and Triple-Color PBNA

**Figure 5:**
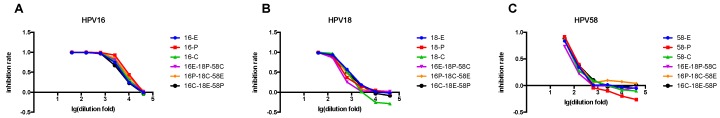
Comparison of single- and three-color PBNAs against HPV16, HPV18, and HPV58. (**A**) Comparison of single- and three-color PBNAs for HPV16; (**B**) Comparison of single- and three-color PBNAs for HPV18; (**C**) Comparison of single- and three-color PBNAs for HPV58. 16-E: HPV16 pseudovirion packaged with the *EGFP* reporter gene; 16-R: HPV16 pseudovirion packaged with the *RFP* reporter gene; 16-C: HPV16 pseudovirion packaged with the *CFP* reporter gene; 16E-18R-58C: HPV16 EGFP + HPV18 RFP + HPV CFP; 16R-18C-58E: HPV16 RFP + HPV18 CFP + HPV58 EGFP; 16C-18E-58R: HPV16 CFP + HPV18 RFP + HPV58 EGFP. The sample tested contained mixed sera from mice immunized with a candidate nonavalent HPV vaccine.

To further improve the throughput of the PBNA, a third color (CFP: far red) was introduced to the assay. Nine kinds of pseudovirions were prepared: HPV16-EGFP, HPV16-RFP, HPV16-CFP, HPV18-EGFP, HPV18-RFP, HPV18-CFP, HPV58-EGFP, HPV58-RFP, and HPV58-CFP. The differently colored pseudovirions and different combinations of pseudovirion were tested against pooled serum samples from mice immunized with a nonavalent (HPV6/11/16/18/31/33/45/52/58) HPV candidate vaccine. The similar inhibition curves suggest that there were no significant differences between the single-color PBNA and triple-color PBNA in testing HPV16, HPV18, and HPV58 (Figure 5).

The single-color and triple-color PBNA were also compared using different kinds of samples, including sera from naturally infected humans, sera from immunized mice, and WHO International Standards for antibodies. For HPV16, HPV18, and HPV58, the coefficients of variation (CVs) were 18–34%, 16–51%, and 28–43%, respectively (Table 1). The HPV16 standard specifically neutralized HPV16 in the triple-color PBNA, with IC_50_ values of 118, 97, and 55, respectively, which are similar to those reported previously [29]. The HPV18 standard specifically neutralized HPV18 in the triple-color PBNA, with IC_50_ values of 801, 1027, and 628, which are similar to those reported previously [14]. Most importantly, no cross-reaction was observed in the triple-color PBNA, confirming the consistency between the triple- and single-color PBNAs.

Because the antibody titers are low in individuals naturally infected with HPV, the agreement between the single- and triple-color PBNAs can be assessed most stringently with this kind of sample. Samples from 48 individuals naturally infected with HPV were used in both assays for HPV16, HPV18, and HPV58, using an IC_50_ of 40 as the cut-off value. The sero-status assignment of the samples with the two assays is cross-tabulated in Table 2. For each of the three HPV genotypes, the agreement rates in qualitative analyses were greater than 95.8% (HPV16, 95.8%; HPV18, 95.8%; and HPV58, 97.9%). The kappa scores between the two assays were 0.92, 0.89, and 0.96 for the three HPV types, respectively, suggesting good agreement between the two assays.

### 3.6. Reproducibility of the Triple-Color PBNA

**Figure 6:**
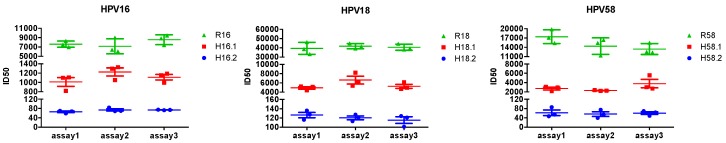
Reproducibility of the triple-color PBNA. R16, R18, and R58 were rabbit serum samples immunized with corresponding mono-valent HPV vaccines. H16.1, H16.2, H18.1, H18.2, H58.1, and H58.2 were serum samples from naturally infected individuals.

To determine the reproducibility of the three-color PBNA, three samples for each type were tested: two from naturally infected individuals (H16.1, H16.2 for HPV16; H18.1, H18.2 for HPV18; H58.1, H58.2 for HPV58) and one from a rabbit immunized three times with the corresponding monovalent HPV vaccines (R16 for HPV16, R18 for HPV18, R58 for HPV58). Each serum was tested nine times in three independent runs (Figure 6). When the serum titers were calculated, the intra-assay and inter-assay coefficients of variation were 2.0%–41.5% and 8.3%–36.2%, respectively, indicating good reproducibility.

### 3.7. Triple-Color PBNA for HPV6-33-45 and HPV11-31-52

**Figure 7:**
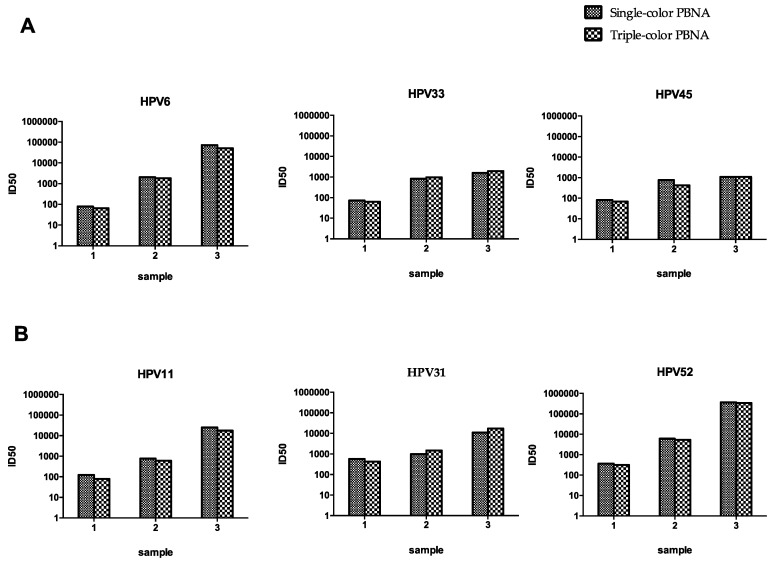
Comparison of the single- and triple-color PBNA for HPV6-33-45 and HPV11-31-52 PBNAs. (**A**) Comparison of the single- and triple-color PBNA for HPV6-33-45; (**B**) Comparison of the single- and triple-color PBNA for HPV11-31-52. Three serum samples from mice immunized with different amounts of the nonavalent vaccines were tested using single-color and triple-color PBNAs, respectively.

A nonavalent HPV vaccine targeting HPV6/11/16/18/31/33/45/52/58 (Gardasil 9; Merck) was approved by the U.S. FDA in 2014 [6]. To test all nine HPV types, another two triple-color pseudovirion mixtures were examined, HPV6-33-45 and HPV11-31-52. Three serum samples from mice immunized with different amounts of the nonavalent vaccines were used to test the consistency between the single-color and triple-color PBNAs. Good concordance was observed between the single- and triple-color PBNAs for both the HPV6-33-45 assay and the HPV11-31-52 assay (Figure 7). The neutralizing antibody profiles were detected for the samples immunized with the nonavalent vaccine with three runs of the assay using the triple-color PBNA.

## 4. Discussion

Neutralizing antibodies are considered the primary protective mechanisms for most prophylactic vaccines, and PBNA is considered the gold standard method for evaluating the immune response raised against HPV vaccines [12,14,30]. However, PBNA is not widely used in clinical trials or sero-epidemiological analysis because it is laborious and complex. To date, a series of optimized PBNAs has been developed and the throughput of PBNAs has been improved to some extent [14]. However, compared with antibody-binding detection assays, especially the multiplex cLIA based on Luminex, the PBNA procedures require considerable simplification. In this study, a new assay based on a fluorescent reporter gene was developed as an economical and easy-to-use method for simultaneously comparing the neutralizing antibodies raised against different HPV types.

FluoroSpot, which is used in the ELISPOT assay, allows the analysis of single cells secreting several cytokines [31]. Here, FluoroSpot directly detected target cells infected with the HPV pseudovirion, without cell digestion or the addition of further substrates. The detection time was less than 5 min for a 96-well plate. The traditional detection strategy for fluorescent proteins is fluorescent microscopy and/or FCM. The results of the EGFP-based pseudovirion infection assay showed good consistency between the FCM and FluoroSpot detection methods. When the single-color method was extended to include multiple colors, any possible cross-interference of the different reporters had to be considered. For example, YFP can be detected by the EGFP wavelength pair. Furthermore, the specificity of the assay decreases with decreasing wavelengths. For example, cell debris and unexpected fibers will present nonspecific blue light at the wavelengths used for the blue fluorescent protein reporter, which is therefore unsuitable for this assay.

Although the mechanisms have not been fully revealed, cross-protection has been observed with bivalent and quadrivalent vaccines [32,33,34]. Therefore, to avoid the detection of false positives, the mixed pseudovirions should be distantly phylogenetically related. HPV16 and HPV18, which belong to the alpha 9 and alpha 7 genotypes, respectively, were selected for the dual-color combination. Based on this principle, closely related HPV types were assigned to different combinations. Therefore, for the immunological analysis of the nonavalent vaccine, mixtures HPV16-18-58, HPV6-33-45, and HPV11-31-52 were used to avoid cross-reaction.

To date, neutralizing methods have been used in few clinical HPV vaccine trials. Instead, antibody binding is used as a surrogate index for the immune response. The coating antigens and principles of detection differ completely between these two types of assays. No comparison data for different clinical trials are available. Placebo controls cannot be used in future clinical studies, especially for the development of second-generation vaccines. Therefore, the development of high-throughput easy-to-use protection-related PBNAs is urgently required for future clinical trials.

## 5. Conclusions

Three triple-color PBNAs, HPV16-18-58, HPV6-33-45, and HPV11-31-52, were developed to evaluate a nonavalent vaccine. This multicolor PBNA provided rapid and precise profiles of the protection-related immune response in vaccine-immunized or naturally infected cohorts. The new assay has advantage over the FCM assay for the easy-to-use procedure, which shortens the detection time from more than 3 h to 5 min for 96-well plate. Even compared to the recently developed high-throughput Gluc-based assay [15], triple-color assay only needs one third of the detection time and sample quantity for the same sample evaluation. With the development of fluorescent reporter proteins and the FluoroSpot technology, more colors for PBNA might become available to improve the analysis of immune responses in the future.

## Figures and Tables

**Table 1:** Comparison of the single- and three-color PBNAs in detecting antibody titers.

		Sample				
	Pseudovirion	A	B	C	D	E
HPV16	16E	113	967	5025	99	<40
	16P	95	1945	8608	109	<40
	16C	75	1225	5786	75	<40
	16E-18P-58C	93	1016	5866	118	<40
	16P-18C-58E	74	1328	7039	97	<40
	16C-18E-58P	<40	761	4381	55	<40
	CV	18%	34%	25%	25%	0%
HPV18	18E	120	16,957	15,630	<40	826
	18P	80	5641	14,820	<40	824
	18C	78	6875	13,912	<40	674
	16E-18P-58C	102	6500	15,366	<40	801
	16P-18C-58E	98	11,296	5106	<40	1067
	16C-18E-58P	98	18,427	9519	<40	628
	CV	16%	51%	34%	0%	19%
HPV58	58E	103	1778	8799	<40	<40
	58P	121	2029	7422	<40	<40
	58C	93	1098	3077	<40	<40
	16E-18P-58C	76	1395	3686	<40	<40
	16P-18C-58E	189	952	10,764	<40	<40
	16C-18E-58P	115	1608	7599	<40	<40
	CV	34%	28%	43%	0%	0%

A, B: Human sera obtained from naturally infected individuals; C: Serum sample from mice immunized with the candidate nonavalent HPV vaccine; D: WHO Anti-HPV16 serum standard (National Institute for Biological Standards and Control (NIBSC) code: 05/134); E: WHO Anti-HPV18 serum standard (NIBSC code: 10/140).

**Table 2:** Agreement between the single- and triple-color PBNAs in a naturally infected cohort.

	Triple-Color PBNA								
Single-Color PBNA	HPV16			HPV18			HPV58		
	−	+	Total	−	+	Total	−	+	Total
−	26	0	26	35	0	35	29	1	30
+	2	20	22	2	11	13	0	18	18
Total	28	16	48	37	11	48	29	19	48
Agreement (%)	95.8			95.8			97.9		
Negative Predictive Value (%)	92.9			94.6			100		
Positive Predictive Value (%)	100			100			94.7		
Kappa score	0.92			0.89			0.96		

## Abbreviations

PBNA: Pseudovirion-based neutralization assay
HPV: Human papillomavirus
US: United States
FCM: Flow cytometry
FDA: Food and Drug Administration
cLIA: Competitive Luminex^®^ immunoassay
VLP: Virus-like particle
VLP-MIA: Virus-like particle-based multiplex immunoassay
GST-L1-MIA: Glutathione S-transferase L1-based MIA
EGFP: Enhanced green fluorescent protein
GLuc: *Gaussia* luciferase
SEAP: Secreted alkaline phosphatase
HEPES: 4-(2-Hydroxyethyl)-1-piperazineethanesulfonic acid
RFP: Red fluorescent protein
E2-CFP: E2-crimson fluorescent protein
WHO: World Health Organization
NIBSC: National institute for Biological Standards and Control
YFP: Yellow fluorescent protein
